# How evolution learns to generalise: Using the principles of learning theory to understand the evolution of developmental organisation

**DOI:** 10.1371/journal.pcbi.1005358

**Published:** 2017-04-06

**Authors:** Kostas Kouvaris, Jeff Clune, Loizos Kounios, Markus Brede, Richard A. Watson

**Affiliations:** 1 ECS, University of Southampton, Southampton, United Kingdom; 2 University of Wyoming, Laramie, Wyoming, United States of America; University of Chicago, UNITED STATES

## Abstract

One of the most intriguing questions in evolution is how organisms exhibit suitable phenotypic variation to rapidly adapt in novel selective environments. Such variability is crucial for evolvability, but poorly understood. In particular, how can natural selection favour developmental organisations that facilitate adaptive evolution in previously unseen environments? Such a capacity suggests foresight that is incompatible with the short-sighted concept of natural selection. A potential resolution is provided by the idea that evolution may discover and exploit information not only about the particular phenotypes selected in the past, but their underlying structural regularities: new phenotypes, with the same underlying regularities, but novel particulars, may then be useful in new environments. If true, we still need to understand the conditions in which natural selection will discover such deep regularities rather than exploiting ‘quick fixes’ (i.e., fixes that provide adaptive phenotypes in the short term, but limit future evolvability). Here we argue that the ability of evolution to discover such regularities is formally analogous to learning principles, familiar in humans and machines, that enable generalisation from past experience. Conversely, natural selection that fails to enhance evolvability is directly analogous to the learning problem of over-fitting and the subsequent failure to generalise. We support the conclusion that evolving systems and learning systems are different instantiations of the same algorithmic principles by showing that existing results from the learning domain can be transferred to the evolution domain. Specifically, we show that conditions that alleviate over-fitting in learning systems successfully predict which biological conditions (e.g., environmental variation, regularity, noise or a pressure for developmental simplicity) enhance evolvability. This equivalence provides access to a well-developed theoretical framework from learning theory that enables a characterisation of the general conditions for the evolution of evolvability.

## Author summary

A striking feature of evolving organisms is their ability to acquire novel characteristics that help them adapt in new environments. The origin and the conditions of such ability remain elusive and is a long-standing question in evolutionary biology. Recent theory suggests that organisms can evolve designs that help them generate novel features that are more likely to be beneficial. Specifically, this is possible when the environments that organisms are exposed to share common regularities. However, the organisms develop robust designs that tend to produce what had been selected in the past and might be inflexible for future environments. The resolution comes from a recent theory introduced by Watson and Szathmáry that suggests a deep analogy between learning and evolution. Accordingly, here we utilise learning theory to explain the conditions that lead to more evolvable designs. We successfully demonstrate this by equating evolvability to the way humans and machines generalise to previously-unseen situations. Specifically, we show that the same conditions that enhance generalisation in learning systems have biological analogues and help us understand why environmental noise and the reproductive and maintenance costs of gene-regulatory connections can lead to more evolvable designs.

## Introduction

### Linking the evolution of evolvability with generalisation in learning systems

Explaining how organisms adapt in novel selective environments is central to evolutionary biology [1–5]. Living organisms are both robust and capable of change. The former property allows for stability and reliable functionality against genetic and environmental perturbations, while the latter provides flexibility allowing for the evolutionary acquisition of new potentially adaptive traits [5–9]. This capacity of an organism to produce suitable phenotypic variation to adapt to new environments is often identified as a prerequisite for *evolvability*, i.e., the capacity for adaptive evolution [7, 10, 11]. It is thus important to understand the underlying variational mechanisms that enable the production of adaptive phenotypic variation [6, 7, 12–18].

Phenotypic variations are heavily determined by intrinsic tendencies imposed by the genetic and the developmental architecture [18–21]. For instance, developmental biases may permit high variability for a particular phenotypic trait and limited variability for another, or cause certain phenotypic traits to co-vary [6, 15, 22–26]. Developmental processes are themselves also shaped by previous selection. As a result, we may expect that past evolution could adapt the distribution of phenotypes explored by future natural selection to amplify promising variations and avoid less useful ones by evolving developmental architectures that are predisposed to exhibit effective adaptation [10, 13]. Selection though cannot favour traits for benefits that have not yet been realised. Moreover, in situations when selection can control phenotypic variation, it nearly always reduces such variation because it favours canalisation over flexibility [23, 27–29].

Developmental canalisation may seem to be intrinsically opposed to an increase in phenotypic variability. Some, however, view these notions as two sides of the same coin, i.e., a predisposition to evolve some phenotypes more readily goes hand in hand with a decrease in the propensity to produce other phenotypes [8, 30, 31]. Kirschner and Gerhart integrated findings that support these ideas under the unified framework of *facilitated variation* [8, 32]. Similar ideas and concepts include the *variational properties* of the organisms [13], the *self-facilitation* of evolution [20] and evolution as *tinkering* [33] and related notions [6, 7, 10, 12]. In facilitated variation, the key observation is that the intrinsic developmental structure of the organisms biases both the amount and the direction of the phenotypic variation. Recent work in the area of facilitated variation has shown that multiple selective environments were necessary to evolve evolvable structures [25, 27, 34–36]. When selective environments contain underlying structural regularities, it is possible that evolution learns to limit the phenotypic space to regions that are evolutionarily more advantageous, promoting the discovery of useful phenotypes in a single or a few mutations [35, 36]. But, as we will show, these conditions do not necessarily enhance evolvability in novel environments. Thus the general conditions which favour the emergence of adaptive developmental constraints that enhance evolvability are not well-understood.

To address this we study the conditions where evolution by natural selection can find developmental organisations that produce what we refer to here as *generalised phenotypic distributions*—i.e., not only are these distributions capable of producing multiple distinct phenotypes that have been selected in the past, but they can also produce novel phenotypes from the same family. Parter et al. have already shown that this is possible in specific cases studying models of RNA structures and logic gates [34]. Here we wish to understand more general conditions under which, and to what extent, natural selection can enhance the capacity of developmental structures to produce suitable variation for selection in the future. We follow previous work on the evolution of development [25] through computer simulations based in gene-regulatory network (GRN) models. Many authors have noted that GRNs share common functionality to artificial neural networks [25, 37–40]. Watson et al. demonstrated a further result, more important to our purposes here; that the way regulatory interactions *evolve* under natural selection is mathematically equivalent to the way neural networks *learn* [25]. During evolution a GRN is capable of learning a memory of multiple phenotypes that were fit in multiple past selective environments by internalising their statistical correlation structure into its ontogenetic interactions, in the same way that learning neural networks store and recall training patterns. Phenotypes that were fit in the past can then be recreated by the network spontaneously (under genetic drift without selection) in the future or as a response to new selective environments that are partially similar to past environments [25]. An important aspect of the evolved systems mentioned above is modularity. Modularity has been a key feature of work on evolvability [6, 29, 41, 42] aiming to facilitate variability that respects the natural decomposable structure of the selective environment, i.e., keep the things together that need to be kept together and separate the things that are independent [6, 12, 20, 41]. Accordingly, the system can perform a simple form of generalisation by separating knowledge from the context in which it was originally observed and re-deploying it in new situations.

Here we show that this functional equivalence between learning and evolution predicts the evolutionary conditions that enable the evolution of generalised phenotypic distributions. We test this analogy between learning and evolution by testing its predictions. Specifically, we resolve the tension between canalisation of phenotypes that have been successful in past environments and anticipation of phenotypes that are fit in future environments by recognising that this is equivalent to prediction in learning systems. Such predictive ability follows simply from the ability to represent structural regularities in previously seen observations (i.e., the training set) that are also true in the yet-unseen ones (i.e., the test set). In learning systems, such generalization is commonplace and not considered mysterious. But it is also understood that successful generalisation in learning systems is not for granted and requires certain well-understood conditions. We argue here that understanding the evolution of development is formally analogous to model learning and can provide useful insights and testable hypotheses about the conditions that enhance the evolution of evolvability under natural selection [42, 43]. Thus, in recognising that learning systems do not really ‘see into the future’ but can nonetheless make useful predictions by generalising past experience, we demystify the notion that short-sighted natural selection can produce novel phenotypes that are fit for previously-unseen selective environments and, more importantly, we can predict the general conditions where this is possible. This functional equivalence between learning and evolution produces many interesting, testable predictions (Table 1).

**Table 1:** Predictions made by porting key lessons of learning theory to evolutionary theory. Confirmed by experiment: † Conditions that facilitate generalised phenotypic distributions, ‡ How generalisation changes over evolutionary time, ◇ Conditions that facilitate generalised phenotypic distributions and ⋆ Sensitivity analysis to parameters affecting phenotypic generalisation.

	Learning Theory	Evolutionary Theory
(a)	Generalisation; ability to produce an appropriate response to novel situations by exploiting regularities observed in past experience (i.e., not rote learning).	Facilitated variation; predisposition to produce fit phenotypes in novel environments (i.e., not just canalisation of past selected targets).†
(b)	The performance of online learning algorithms (i.e., processing one training example at a time) are learning-rate dependent. Both high and low learning rates can lead to situations of under-fitting; failure of the learning system to capture the regularities of the training data [51].	The evolution of generalised phenotypic distributions is dependent on the time-scale of environmental switching. Both high and low time-scales can lead to inflexible developmental structures that fail to capture the functional dependencies of the past phenotypic targets.◇
(c)	The problem of over-fitting: improved performance on the training set comes at the expense of generalisation performance on the test set. Over-fitting occurs when the model learns to focus on idiosyncrasies or noise in the training set [52]. Accordingly, the model starts learning the particular irrelevant relationships existing in the training examples rather than the ‘true’ underlying relationships that are relevant to the general class. This leads to memorisation of specific training examples, which decreases the ability to generalize, and thus perform well, on new data.	Failure of natural selection to evolve generalised developmental organisations: improved average fitness gained by decreasing the phenotypic variation of descendants comes at the expense of potentially useful variability for future selective environments. Favouring immediate fitness benefits would lead to robust developmental structures that canalise the production of the selected phenotypes in the current selective environment. Yet, this sets up a trade-off between robustness and evolvability, since natural selection would always favour inflexible developmental organisations that reduce phenotypic variability and thus hinder the discovery of useful phenotypes that can have fitness benefits in the future.‡
(d)	Conditions that alleviate the problem of over-fitting: (1) training with noisy data, i.e., adding noise during the learning phase (jittering), (2) regularisation (parsimony pressure), i.e., introducing a connection cost term into the objective function that favours connections of small values (*L*_2_-regularisation) or fewer connections (*L*_1_-regularisation).	Evolutionary conditions that facilitate the evolution of generalised phenotypic distributions, and thus evolvability: (1) extrinsic noise in selective environments, (2) direct selection pressure on the cost of ontogenetic interactions, which favour simpler developmental processes and sparse network structures.†‡
(e)	*L*_2_-regularisation results in similar behaviour as early stopping; an ad-hoc technique that prevents over-fitting by stopping learning when over-fitting begins [51].	Favouring weak connectivity via connection costs results in similar behaviour as stopping adaptation at an early stage.†‡.
(f)	Training with noise results in similar behaviour to *L*_2_-regularisation [51].	Noisy environments can enhance the evolution of generalised developmental organisation in a similar manner as favouring weak connectivity.†‡.
(g)	Generalisation performance is dependent on the appropriate level of regularisation and the level of noise, i.e., it depends on the inductive biases, or prior assumptions about which models are more likely to be correct, such as a priori perference for simple models via parsimony pressures.	The evolution of generalised phenotypic distributions is dependent on the strength of selection pressure on the cost of connections and the level of environmental noise.⋆
(h)	*L*_1_-regularisation results in better generalisation performance than *L*_2_-regularisation in problems with simple modularity/independent features.	Favouring sparsity results in more evolvable developmental structures than favouring weak connectivity for modularly varying environments with weak or unimportant inter-modular dependencies.†‡

In particular, the following experiments show that techniques that enhance generalisation in machine learning correspond to evolutionary conditions that facilitate generalised phenotypic distributions and hence increased evolvability. Specifically, we describe how well-known machine learning techniques, such as learning with noise and penalising model complexity, that improve the generalisation ability of learning models have biological analogues and can help us understand how noisy selective environments and the direct selection pressure on the reproduction cost of the gene regulatory interactions can enhance evolvability in gene regulation networks. This is a much more sophisticated and powerful form of generalisation than previous notions that simply extrapolate previous experience. The system does not merely extend its learned behaviour outside its past ‘known’ domain. Instead, we are interested in situations where the system can create new knowledge by discovering and systematising emerging patterns from past experience, and more notably, how the system separates that knowledge from the context in which it was originally observed, so that it can be re-deployed in new situations.

Some evolutionary mechanisms and conditions have been proposed as important factors for improved evolvability. Some concern the modification of genetic variability (e.g., [36, 44, 45] and [46]), while others concern the nature of selective environments and the organisation of development including multiple selective environments [36], sparsity [47], the direct selective pressure on the cost of connections (which can induce modularity [27, 44] and hierarchy [48]), low developmental biases and constraints [49] and stochasticity in GRNs [50]. In this paper, we focus on mechanisms and conditions that can be unified and better understood in machine learning terms, and more notably, how we can utilise well-established theory in learning to characterise general conditions under which evolvability is enhanced. We thus provide the first theory to characterise the general conditions that enhance the evolution of developmental organisations that generalise information gained from past selection, as required to enhance evolvability in novel environments.

### Experimental setup

The main experimental setup involves a non-linear recurrent GRN which develops an embryonic phenotypic pattern, *G*, into an adult phenotype, *P*_*a*_, upon which selection can act [25]. An adult phenotype represents the gene expression profile that results from the dynamics of the GRN. Those dynamics are determined by the gene regulatory interactions of the network, *B* [38, 39, 47, 53, 54] (see Developmental Model in S1 Appendix). We evaluate the fitness of a given genetic structure based on how close the developed phenotype is to the target phenotypic pattern, *S*. *S* characterises the direction of selection for each phenotypic trait, i.e., element of gene expression profile, in the current environment. The dynamics of selective environments are modelled by switching from one target phenotype to another every *K* generations. *K* is chosen to be considerably smaller than the overall number of generations simulated. Below, we measure evolutionary time in *epochs*, where each epoch denotes *N*_*T*_ × *K* generations and *N*_*T*_ corresponds to the number of target phenotypes. (Note that *epoch* here is a term we are borrowing from machine learning and does not represent geological timescale.)

**Fig 1:**
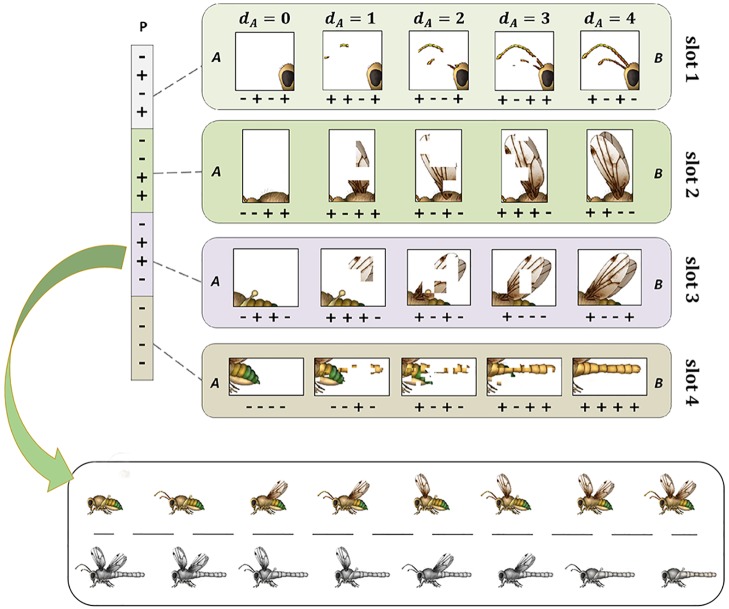
Pictorial representation of phenotypes. (Top) Schematic representation of mapping from phenotypic pattern sequences onto pictorial features. Each phenotypic ‘slot’ represents a set of features (here 4) controlling a certain aspect of the phenotype (e.g., front wings, halteres and antennae). Within the possible configurations in each slot (here 16), there are two particular configurations (state A and B) that are fit in some environment or another (see Developmental Model in S1 Appendix). For example, ‘+ + −−’ in the second slot (from the top, green) of the phenotypic pattern encodes for a pair of front wings (state B), while ‘− − ++’ encodes for their absence (state A). States A and B are the complement of one another, i.e., not neighbours in phenotype space. All of the other intermediate states (here 14) are represented by a random mosaic image of state A and B, based on their respective distance. *d*_*A*_ indicates the Hamming distance between a given state and state A. Accordingly, there exist $\left(\begin{array}{c} 4\\ d_{A} \end{array}\right)$ potential intermediate states (i.e., 4 for *d*_*A*_ = 1, 6 for *d*_*A*_ = 2 and 4 for *d*_*A*_ = 3). (Bottom) Pictorial representation of all phenotypes that are perfectly adapted to each of eight different environments. Each target phenotype is analogous to an insect-like organism comprised of 4 functional features. The grey phenotypic targets correspond to bit-wise complementary patterns of the phenotypes on the top half of the space. For example, in the rightmost, top insect, the antennae, forewings, and hindwings are present, and the tail is not. In the rightmost, bottom insect (the bitwise complement of the insect above it), the antennae, forewings, and hindwings are absent, but the tail is present. We define the top row as ‘the class’ and we disregard the bottom complements as degenerate forms of generalisation.

**Fig 2:**
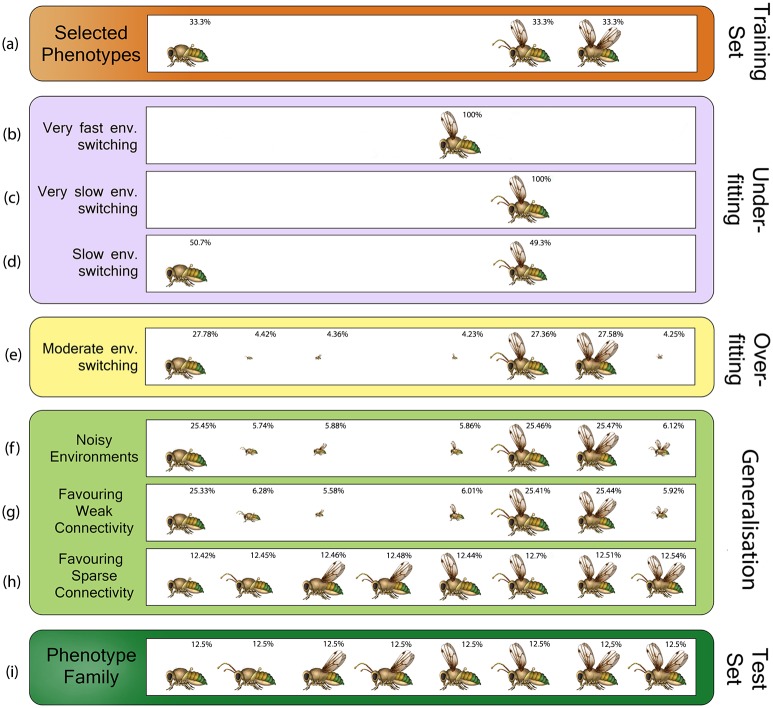
Conditions that facilitate generalised phenotypic distributions. Potential phenotypic distributions induced by the evolved developmental process under 1) different time-scales of environmental switching, 2) environmental noise (*κ* = 35 × 10^−4^) and 3) direct selection pressure for weak (*λ* = 38) and sparse connectivity (*λ* = 0.22). The organisms were exposed to three selective environments (a) from the general class (i). Developmental memorisation of past phenotypic targets clearly depends on the time-scale of environmental change. Noisy environments and parsimony pressures enhance the generalisation ability of development predisposing the production of previously unseen targets from the class. The size of the insect-like creatures describes relative frequencies and indicates the propensity of development to express the respective phenotype (phenotypes with frequency less than 0.01 were ignored). Note that the initial developmental structure represented all possible phenotypic patterns equally (here 2^12^ possible phenotypes).

In the following experiments all phenotypic targets are chosen from the same class (as in [25, 34]). This class consists of 8 different modular patterns that correspond to different combinations of sub-patterns. Each sub-pattern serves as a different function as pictorialised in Fig 1. This modular structure ensures that the environments (and thus the phenotypes that are fittest in those environments) share common regularities, i.e., they are all built from different combinations from the same set of modules. We can then examine whether the system can actually ‘learn’ these systematicities from a limited set of examples and thereby generalise from these to produce novel phenotypes within the same class. Our experiments are carried out as follows. The population is evolved by exposure to a limited number of selective environments (training). We then analyse conditions under which new phenotypes from the same family are produced (test). As an exemplary problem we choose a training set comprised of three phenotypic patterns from the class (see Fig 2a).

One way to evaluate the generalisation ability of developmental organisations is to evolve a population to new selective environments and evaluate the evolved predisposition of the development system to produce suitable phenotypes for those environments (as per [34]). We do this at the end of experimental section. We also use a more stringent test and examine the spontaneous production of such phenotypes induced by development from random genetic variation. Specifically, we examine what phenotypes the evolved developmental constraints and biases *B* are predisposed to create starting from random initial gene expression levels, *G*. For this purpose, we perform a post-hoc analysis. First, we estimate the phenotypic distributions induced by the evolved developmental architecture under drift. Since mutation on the direct effects on the embryonic phenotypes (*G*) in this model is much greater than mutation on regulatory interactions (*B*) (see Methods), we estimate drift with a uniformly random distribution over *G* (keeping *B* constant). Then we assess how successful the evolved system is at producing high-fitness phenotypes, by seeing if the phenotypes produced by the evolved correlations, *B*, tend to be members of the general class (see Methods).

## Results and discussion

### Conditions that facilitate generalised phenotypic distributions

In this section, we focus on the conditions that promote the evolution of adaptive developmental biases that facilitate generalised variational structures. To address this, we examine the distributions of potential phenotypic variants induced by the evolved developmental structure in a series of different evolutionary scenarios: 1) different time-scales of environmental switching, 2) environmental noise and 3) direct selection pressure for simple developmental processes applied via a the cost of ontogenetic interactions favouring i) weak and ii) sparse connectivity.

#### Rate of environmental switching (learning rates)

In this scenario, we assess the impact of the rate at which selective environments switch on the evolution of generalised developmental organisations. This demonstrates prediction (b) from Table 1. The total number of generations was kept fixed at 24 × 10^6^, while the switching intervals, *K*, varied. In all reproductive events, *G* is mutated by adding a uniformly distributed random value drawn in [−0.1, 0.1]. Additionally, in half the reproduction events, all interaction coefficients are mutated slightly by adding a uniformly distributed value drawn from [−0.1/(15*N*^2^), 0.1/(15*N*^2^)], where *N* corresponds to the number of phenotypic traits.

Prior work on facilitated variation has shown that the evolution of evolvability in varying selective environments is dependent on the time-scale of environmental change [34–36]. This is analogous to the sensitivity of generalisation to learning rate in learning systems. The longer a population is exposed to a selective environment, the higher the expected adaptation accumulated to that environment would be. Accordingly, the rate of change in a given environment (learning rate) can be controlled by the rate of environmental change (sample rate). Slow and fast environmental changes thus correspond to fast and slow learning rates respectively.

We find that when the environments rapidly alternated from one to another (e.g., *K* = 2), natural selection canalised a single phenotypic pattern (Fig 2b). This phenotype however did not correspond to any of the previously selected ones (Fig 2a). Rather, this corresponds to the combination of phenotypic characters that occurs most in each of the seen target phenotypes. Hence, it does best on average over the past selective environments. For example, over the three patterns selected in the past it is more common that halteres are selected than a pair of back wings, or a pair of front wings is present more often than not and so on.

When environments changed very slowly (e.g., *K* = 4 × 10^6^), development canalised the first selective environment experienced, prohibiting the acquisition of any useful information regarding other selective environments (Fig 2c). The situation was improved for a range of slightly faster environmental switching times (e.g., *K* = 2 × 10^6^), where natural selection also canalised the second target phenotype experienced, but not all three (Fig 2d). Canalisation can therefore be opposed to evolvability, resulting in very inflexible models that fail to capture any or some of the relevant regularities in the past or current environments, i.e., *under-fitting*. Such developmental organisations could provide some limited immediate fitness benefits in the short-term, but are not good representatives of either the past, or the general class.

When the rate of environmental switching was intermediate (e.g., *K* = 4 × 10^4^), the organisms exhibited developmental memory [25]. Although initially all possible phenotypic patterns (here 2^12^) were equally represented by development, the variational structure of development was adapted over evolutionary time to fit the problem structure of that past, by canalising the production of previously seen targets (Fig 2e, see also Fig B in Supporting Figures in S1 Appendix). This holds for a wide range of intermediate switching intervals (see Fig C in Supporting Figures in S1 Appendix). This observations illustrates the ability of evolution to genetically acquire and utilise information regarding the statistical structure of previously experienced environments.

The evolved developmental constraints also exhibited generalised behaviour by allowing the production of three additional phenotypes that were not directly selected in the past, but share the same structural regularities with the target phenotypes. These new phenotypic patterns correspond to novel combinations of previously-seen phenotypic features. Yet, the propensity to express these extra phenotypes was still limited. The evolved variational mechanism over-represented past targets, failing to properly generalise to all potential, but yet-unseen selective environments from the same class as the past ones, i.e., over-fitted (see below). We find no rate of environmental variation capable of causing evolution by natural selection to evolve a developmental organisation that produces the entire class. Consequently, the rate of environmental change can facilitate the evolution of developmental memory, but does not always produce good developmental generalisation.

**Fig 3:**
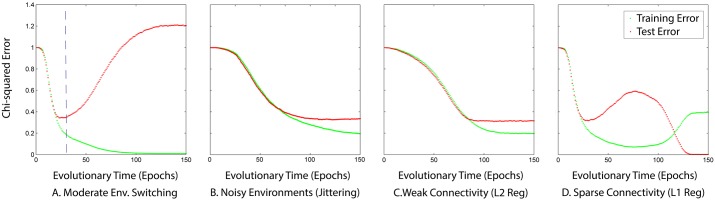
How generalisation changes over evolutionary time. The match between phenotypic distributions generated by evolved GRN and the target phenotypes of selective environments the developmental system has been exposed to (training error) and all selective environments (test error) against evolutionary time for (A) moderate environmental switching, (B) noisy environments, (C) favouring weak connectivity and (D) favouring sparse connectivity. The vertical dashed line denotes when the ad-hoc technique of early stopping would be ideal, i.e. at the moment the problem of over-fitting begins. Favouring weak connectivity and jittering exhibits similar effects on test error as applying early stopping.

Here we argue that the problem of natural selection failing to evolve generalised phenotypic distributions in certain cases is formally analogous to the problem of learning systems failing to generalise due to either under- or over-fitting. In learning, under-fitting is observed when a learning system is incapable of capturing a set of exemplary observations. On the other hand, over-fitting is observed when a model is over-trained and memorises a particular set of exemplary observations, at the expense of predictive performance on previously unseen data from the class [51]. Over-fitting occurs when the model learns to focus on idiosyncrasies or noise in the training set [52]. Similarly, canalisation to past selective environments can be opposed to evolvability if canalised phenotypes from past environments are not fit in future environments. Specifically, canalisation can be opposed to evolvability by either 1) (first type of underfitting, from high learning rates) reducing the production of all phenotypic characters except those that are fit in the selective environments that happen to come early (Fig 2c), 2) (second type of under-fitting, from low learning rates) reducing the production of all characters except those that are fit on average over the past selective environments (Fig 2b), or 3) (over-fitting) successfully producing a sub-set of or all phenotypes that were fit in the past selective environments, but inhibiting the production of new and potentially useful phenotypic variants for future selective environments (Fig 2d and 2e).

Below, we investigate the conditions under which an evolutionary process can avoid canalising the past and remain appropriately flexible to respond to novel selective environments in the future. To do so, we test whether techniques used to avoid under-fitting and over-fitting that improve generalisation to unseen test sets in learning models will likewise alleviate canalisation to past phenotypic targets and improve fit to novel selective environments in evolutionary systems. For this purpose, we choose the time scale of environmental change to be moderate (*K* = 20000). This constitutes our control experiment in the absence of environmental noise and/or any selective pressure on the cost of connections. In the following evolutionary scenarios, simulations were run for 150 epochs. This demonstrates prediction d,e, and f from Table 1.

#### Noisy environments (training with noisy data)

In this scenario, we investigate the evolution of generalised developmental organisations in noisy environments by adding Gaussian noise, *n*_*μ*_ ∼ *N*(0, 1) to the respective target phenotype, *S*, at each generation. The level of noise was scaled by parameter *κ*. In order to assess the potential of noisy selection to facilitate phenotypic generalisation, we show results for the optimal amount of noise (here *κ* = 35 × 10^−4^). Later, we will show how performance varies with the amount of noise.

We find that the distribution of potential phenotypic variants induced by the evolved development in noisy environments was still biased in generating past phenotypic patterns (Fig 2f). However, it slightly improved fit to other selective environments in the class compared with Fig 2e. The evolved developmental structure was characterised by more suitable variability, displaying higher propensity, compared to the control, in producing those variants from the class that were not directly selected in the past.

Masking spurious details in the training set by adding noise to the training samples during the training phase is a general method to combat the problem of over-fitting in learning systems. This technique is known as ‘training with noise’ or ‘jittering’ [51] and is closely related to the use of intrinsic noise in deep neural networks; a technique known as ‘dropout’ [55]. The intuition is that when noise is applied during the training phase, it makes it difficult for the optimisation process to fit the data precisely, and thus it inhibits capturing the idiosyncrasies of the training set. Training with noise is mathematically equivalent to a particular way of controlling model complexity known as Tikhonov regularisation [51].

#### Favouring weak connectivity (*L*_2_-regularisation)

In this scenario, the developmental structure was evolved under the direct selective pressure for weak connectivity—favouring regulatory interactions of small magnitude, i.e., *L*_2_-regularisation (see Methods). Weak connectivity is achieved by applying a direct pressure on the cost of connections that is proportional to their magnitude. This imposes constraints on the evolution of the model parameters by penalising extreme values.

Under these conditions natural selection discovered more general phenotypic distributions. Specifically, developmental generalisation was enhanced in a similar manner as in the presence of environmental noise, favouring similar weakly generalised phenotypic distributions. The distribution of potential phenotypic variants induced by development displayed higher propensity in producing useful phenotypic variants for potential future selective environments (Fig 2g).

#### Favouring sparse connectivity (*L*_1_-regularisation)

In this scenario, the developmental structure was evolved under the direct selective pressure for sparse connectivity—favouring fewer regulatory interactions, i.e., *L*_1_-regularisation. Sparse connectivity is achieved by applying an equal direct pressure on the cost of connections. This imposes constraints on the evolution of the parameters by decreasing all non-zero values equally, and thus favouring models using fewer connections.

We find that under these conditions the evolution of generalised developmental organisations was dramatically enhanced. The evolved phenotypic distribution (Fig 2h) was a perfect representation of the class (Fig 2i). We see that the evolved developmental process under the pressure for sparsity favoured the production of novel phenotypes that were not directly selected in the past. Those novel phenotypes were not arbitrary, but characterised by the time-invariant intra-modular regularities common to past selective environments. Although the developmental system was only exposed to three selective environments, it was able to generalise and produce all of the phenotypes from the class by creating novel combinations of previously-seen modules. More notably, we see that the evolved developmental process also pre-disposed the production of that phenotypic pattern that was missing under the conditions for weak connectivity and environmental noise due to strong developmental constraints.

Moreover, the parsimonious network topologies we find here arise as a consequence of a direct pressure on the cost of connections. The hypothesis that sparse network can arise through a cost minimisation process is also supported by previous theoretical findings advocating the advantages of sparse gene regulation networks [56]. Accordingly, natural selection favours the emergence of gene-regulatory networks of minimal complexity. In [56], Leclerc argues that sparser GRNs exhibit higher dynamical robustness. Thus, when the cost of complexity is considered, robustness also implies sparsity. In this study, however, we demonstrated that sparsity gives rise to enhanced evolvability. This indicates that parsimony on the connectivity of the GRNs is a property that may facilitate both robustness and evolvability.

Favouring weak or sparse connectivity belongs in a general category of *regularisation* methods that alleviate over-fitting by penalising unnecessary model complexity via the application of a parsimony pressure that favours simple models with fewer assumptions on the data, i.e., imposing a form of Occam’s razor on solutions (e.g., the Akaike [57] and [58] Bayesian information criteria, limiting the number of features in decision trees [59], or limiting the tree depth in genetic programming [60]). The key observation is that networks with too few connections will tend to under-fit the data (because they are unable to represent the relevant interactions or correlations in the data); whereas networks with more connections than necessary will tend to over-fit the idiosyncrasies of the training data, because they can memorize those idiosyncrasies instead of being forced to learn the underlying general pattern.

### How generalisation changes over evolutionary time

We next asked why costly interactions and noisy environments facilitate generalised developmental organisations. To understand this, we monitor the match between the phenotypic distribution induced by the evolved developmental process and the ones that describe the past selective environments (training set) and all potential selective environments (test set) respectively over evolutionary time in each evolutionary setting (see Methods). Following conventions in learning theory, we term the first measure ‘training error’ and the second ‘test error’. This demonstrates predictions c, e and f from Table 1.

The dependence of the respective errors on evolutionary time are shown in Fig 3. For the control scenario (panel A) we observe the following trend. Natural selection initially improved the fit of the phenotypic distributions to both distributions of past and future selective environments. Then, while the fit to past selective environments continued improving over evolutionary time, the fit to potential, but yet-unseen, environments started to deteriorate (see also Fig B in Supporting Figures in S1 Appendix). The evolving organisms tended to accurately *memorise* the idiosyncrasies of their past environments, at the cost of losing their ability to retain appropriate flexibility for the future, i.e., over-fitting. The dashed-line in Fig 3A indicates when the problem of over-fitting begins, i.e., when the test error first increases. We see that canalisation can be opposed to the evolution of generalised phenotypic distributions in the same way over-fitting is opposed to generalisation. Then, we expect that preventing the canalisation of past targets can enhance the generalisation performance of the evolved developmental structure. Indeed, Fig 3B, 3C and 3D confirm this hypothesis (predictions a-c from Table 1).

In the presence of environmental noise, the generalisation performance of the developmental structure was improved by discovering a set of regulatory interactions that corresponds to the minimum of the generalisation error curve of 0.34 (Fig 3B). However, natural selection in noisy environments was only able to postpone canalisation of past targets and was unable to avoid it in the long term. Consequently, stochasticity improved evolvability by decreasing the speed at which over-fitting occurs, allowing for the developmental system to spend more time at a state which was characterised by high generalisation ability (see also Fig A in The Structure of Developmental Organisation in S1 Appendix). On the other hand, under the parsimony pressure for weak connectivity, the evolving developmental system maintained the same generalisation performance over evolutionary time. The canalisation of the selected phenotypes was thus prevented by preventing further limitation of the system’s phenotypic variability. Note that the outcome of these two methods (Fig 3B and 3C) resembles in many ways the outcome as if we stopped at the moment when the generalisation error was minimum, i.e., early stopping; an ad-hoc solution to preventing over-fitting [51]. Accordingly, learning is stopped before the problem of over-fitting begins (see also Fig A in The Structure of Developmental Organisation in S1 Appendix). Under parsimony pressure for sparse connectivity, we observe that the generalisation error of the evolving developmental system reached zero (Fig 3D). Accordingly, natural selection successfully exploited the time-invariant regularities of the environment properly representing the entire class (Fig 2h). Additionally, Fig D in Supporting Figures in S1 Appendix shows that the entropy of the phenotypic distribution reduces as expected over evolutionary time as the developmental process increasingly canalises the training set phenotypes. In the case of perfect generalisation to the class (sparse connectivity), this convergence reduces from 16 bits (the original phenotype space) to four bits, corresponding to four degrees of freedom where each of the four modules vary independently. In the other cases, overfitting is indicated by reducing to less than four bits.

### Sensitivity analysis to parameters affecting phenotypic generalisation

As seen so far, the generalisation ability of development can be enhanced under the direct selective pressure for both sparse and weak connectivity and the presence of noise in the selective environment, when the strength of parsimony pressure and the level of noise were properly tuned. Different values of *λ* and *κ* denote different evolutionary contexts, where *λ* determines the relative burden placed on the fitness of the developmental system due to reproduction and maintenance of its elements, or other physical constraints and limitations, and *κ* determines the amount of extrinsic noise found in the selective environments (see Evaluation of fitness).

**Fig 4:**
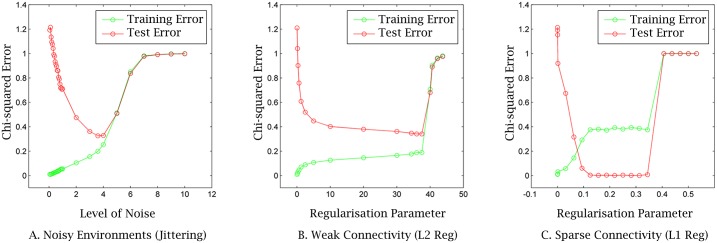
Role of the strength of parsimony pressure and the level of environmental noise. The match between phenotypic distributions and the selective environments the network has been exposed to (training error) and all possible selective environments of the same class (generalisation error) for (A) noisy environments against parameter *κ* and under the parsimony pressure weak (B) and sparse (C) connectivity against parameter *λ*.

In the following, we analyse the impact of the strength of parsimony pressure and the level of environmental noise on the evolution of generalised developmental organisations. Simulations were run for various values of parameters *λ* and *κ*. Then, the training and generalisation error were evaluated and recorded (Fig 4). This demonstrates prediction (g) from Table 1.

We find that in the extremes, low and high levels of parsimony pressures, or noise, gave rise to situations of over-fitting and under-fitting respectively (Fig 4). Very small values of *λ*, or *κ*, were insufficient at finding good regulatory interactions to facilitate high evolvability to yet-unseen environments, resulting in the canalisation of past targets, i.e., over-fitting. On the other hand, very large values of *λ* over-constrained the search process hindering the acquisition of any useful information regarding environment’s causal structure, i.e., under-fitting. Specifically, with a small amount of *L*_1_-regularisation, the generalisation error is dropped to zero. This outcome holds for a wide spectrum of the regularisation parameter *ln*(*λ*) ∈ [0.15, 0.35]. However, when *λ* is very high (here *λ* = 0.4), the selective pressure on the cost of connection was too large; this resulted in the training and the generalisation errors corresponds to the original ‘no model’ situation (Fig 4C). Similarly, with a small amount of *L*_2_-regularisation, the generalisation error quickly drops. In the range [10, 38] the process became less sensitive to changes in *λ*, resulting in one optimum at *λ* = 38 (Fig 4B). Similar results were also obtained for jittering (Fig 4A). But the generalisation performance of the developmental process changes ‘smoothly’ with *κ*, resulting in one optimum at *κ* = 35 × 10^−4^ (Fig 4A). Inductive biases need to be appropriate for a given problem, but in many cases a moderate bias favouring simple models is sufficient for non-trivial generalisation.

### Generalised developmental biases improve the rate of adaptation

Lastly we examine whether generalised phenotypic distributions can actually facilitate evolvability. For this purpose, we consider the rate of adaptation to each of all potential selective environments as the number of generations needed for the evolving entities to reach the respective target phenotype.

To evaluate the propensity of the organisms to reach a target phenotype as a systemic property of its developmental architecture, the regulatory interactions were kept fixed, while the direct effects on the embryonic phenotype were free to evolve for 2500 generations, which was empirically found to be sufficient for the organisms to find a phenotypic target in each selective environment (when that was allowed by the developmental structure). In each run, the initial gene expression levels were uniformly chosen at random. The results here were averaged over 1000 independent runs, for each selective environment and for each of the four different evolutionary scenarios (as described in the previous sections). Then, counts of the average number of generations to reach the target phenotype of the corresponding selective environment were taken. This was evaluated by measuring the first time the developmental system achieved maximum fitness possible. If the target was not reached, the maximum number of generations 2500 was assigned.

**Fig 5:**
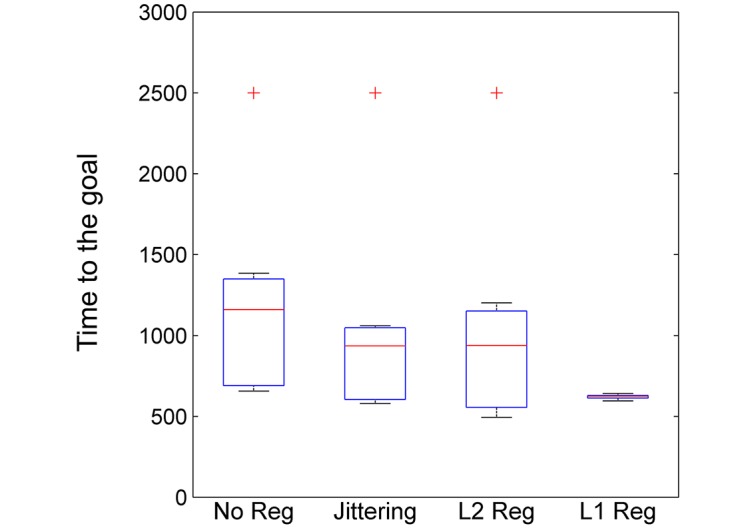
Generalised developmental organisations improve the rate of adaptation to novel selective environments. Boxplot of the generations taken for the evolved developmental systems to reach the target phenotype for all potential selective environments under different evolutionary conditions. The developmental architecture is kept fixed and only the direct effects on the embryonic phenotype are free to evolve. Organisms that facilitate generalised phenotypic distributions, such as the ones evolved in noisy environments or under the direct pressure on the cost connections, adapt faster to novel selective environments exhibiting enhanced evolvability. The outliers indicate the inability of the corresponding evolved developmental structures to reach that selective target due to strong developmental constraints.

We find that organisms with developmental organisations evolved in noisy environments or the parsimony pressure on the cost of connections adapted faster than the ones in the control scenario (Fig 5). The outliers in the evolutionary settings of moderate environmental switching, noisy environments and favouring weak connectivity, indicate the inability of the developmental system to express the target phenotypic pattern for that selective environment due to the strong developmental constraints that evolved in those conditions. This corresponds to the missing phenotype from the class we saw above in the evolved phenotypic distributions induced by development (Fig 2e, 2f and 2g). In all these three cases development allowed for the production of the same set of phenotypic patterns. Yet, developmental structures evolved in the presence of environmental noise or under the pressure for weak connectivity exhibited higher adaptability due to their higher propensity to produce other phenotypes of the structural family. In particular, we see that for the developmental process evolved under the pressure for sparsity, the rate of adaptation of the organisms was significantly improved. The variability structure evolved under sparsity to perfectly represent the functional dependencies between phenotypic traits. Thus, it provided a selective advantage guiding phenotypic variation in more promising directions.

### Conclusions

The above experiments demonstrated the transfer of predictions from learning models into evolution, by specifically showing that: a) the evolution of generalised phenotypic distributions is dependent on the time-scale of environmental switching, in the same way that generalisation in online learning algorithms is learning-rate dependent, b) the presence of environmental noise can be beneficial for the evolution of generalised phenotypic distributions in the same way training with corrupted data can improve the generalisation performance of learning systems with the same limitations, c) direct selection pressure for weak connectivity can enhance the evolution of generalised phenotypic distributions in the same way *L*_2_-regularisation can improve the generalisation performance in learning systems, d) noisy environments result in similar behaviour as favouring weak connectivity, in the same way that Jittering can have similar effects to *L*_2_-regularisation in learning systems, e) direct selection pressure for sparse connectivity can enhance the evolution of generalised phenotypic distributions in the same way that *L*_1_-regularisation can improve the generalisation performance in learning systems, f) favouring weak connectivity (i.e., *L*_2_-regularisation) results in similar behaviour to early stopping, g) the evolution of generalised phenotypic distributions is dependent on the strength of selection pressure on the cost of connections and the level of environmental noise, in the same way generalisation is dependent on the level of inductive biases and h) in simple modularly varying environments with independent modules, sparse connectivity enhances the generalisation of phenotypic distributions better than weak connectivity, in the same way that in problems with independent features, *L*_1_-regularisation results in better generalisation than *L*_2_-regularisation.

Learning is generally *contextual*; it gradually builds upon what *concepts* are already known. Here these concepts correspond to the repeated modular sub-patterns persisting over all observations in the training set which become encoded in the modular components of the evolved network. The inter-module connections determine which combinations of (sub-)attractors in each module are compatible and which are not. Therefore, the evolved network representation can be seen as dictating a higher-order conceptual (combinatorial) space based on previous experience. This enables the evolved developmental system to explore permitted combinations of features constrained by past selection. Novel phenotypes can thus arise through new combinations of previously selected phenotypic features explicitly embedded in the developmental architecture of the system [25]. Indeed, under the selective pressure for sparse connectivity, we observe that the phenotypic patterns generated by the evolved developmental process consisted of combinations of features from past selected phenotypic patterns. Thus, we see that the ‘developmental memories’ are stored and recalled in combinatorial fashion allowing generalisation.

We see that noisy environments and the parsimony pressure on the cost of connections led to more evolvable genotypes by internalising more general models of the environment into their developmental organisation. The evolved developmental systems did not solely capture and represent the specific idiosyncrasies of past selective environments, but internalised the regularities that remained time-invariant in all environments of the given class. This enabled natural selection to ‘anticipate’ novel situations by accumulating information about and exploiting the tendencies in that class of environments defined by the regularities. Peculiarities of past targets were generally represented by weak correlations between phenotypic characters as these structural regularities were not typically present in all of the previously-seen selective environments. Parsimony pressures and noise then provided the necessary selective pressure to neglect or de-emphasise such spurious correlations and maintain only the strong ones which tended to correspond to the underlying problem structure (in this case, the intra-module correlations only, allowing all combinations of fit modules). More notably, we see that the parsimony pressure for sparsity favoured more evolvable developmental organisations that allowed for the production of a novel and otherwise inaccessible phenotype. Enhancing evolvability by means of inductive biases is not for granted in evolutionary systems any more than such methods have guarantees in learning systems. The quality of the method depends on information about past targets and the strength of the parsimony pressure. Inductive biases can however constrain phenotypic evolution into more promising directions and exploit systematicities in the environment when opportunities arise.

In this study we demonstrated that canalisation can be opposed to evolvability in biological systems the same way under- or over-fitting can be opposed to generalisation in learning systems. We showed that conditions that are known to alleviate over-fitting in learning are directly analogous to the conditions that enhance the evolution of evolvability under natural selection. Specifically, we described how well-known techniques, such as learning with noise and penalising model complexity, that improve the generalisation ability of learning models can help us understand how noisy selective environments and the direct selection pressure on the reproduction cost of the gene regulatory interactions can enhance context-specific evolvability in gene regulation networks. This opens-up a well-established theoretical framework, enabling it to be exploited in evolutionary theory. This equivalence demystifies the basic idea of the evolution of evolvability by equating it with generalisation in learning systems. This framework predicts the conditions that will enhance generalised phenotypic distributions and evolvability in natural systems.

## Methods

### Evolution of GRNs

We model the evolution of a population of GRNs under strong selection and weak mutation where each new mutation is either fixed or lost before the next arises. This emphasises that the effects we demonstrate do not require lineage-level selection [61–63]—i.e., they do not require multiple genetic lineages to coexist long enough for their mutational distributions to be visible to selection. Accordingly a simple hill-climbing model of evolution is sufficient [25, 36].

The population is represented by a single genotype [*G*, *B*] (the direct effects and the regulatory interactions respectively) corresponding to the average genotype of the population. Similarly, mutations in *G* and *B* indicate slight variations in population means. Consider that *G*′ and *B*′ denote the respective mutants. Then the adult mutant phenotype, $P_{a}^{\prime }$, is the result of the developmental process, which is characterised by the interaction *B*′, given the direct effects *G*′. Subsequently, the fitness of *P*_*a*_ and $P_{a}^{\prime }$ are calculated for the current selective environment, *S*. If $f_{S}\left(P_{a}^{\prime }\right)>f_{S}\left(P_{a}\right)$, the mutation is beneficial and therefore adopted, i.e., *G*_*t*+1_ = *G*′ and *B*_*t*+1_ = *B*′. On the other hand, when a mutation is deleterious, *G* and *B* remain unchanged.

The variation on the direct effects, *G*, occurs by applying a simple point mutation operator. At each evolutionary time step, *t*, an amount of *μ*_1_ mutation, drawn from [−0.1, 0.1] is added to a single gene *i*. Note that we enforce all *g*_*i*_ ∈ [−1, 1] and hence the direct effects are hard bounded, i.e., *g*_*i*_ = *min*{*max*{*g*_*i*_ + *μ*_1_, −1}, 1}. For a developmental architecture to have a meaningful effect on the phenotypic variation, the developmental constraints should evolve considerably slower than the phenotypic variation they control. We model this by setting the rate of change of *B* to lower values as that for *G*. More specifically, at each evolutionary time step, *t*, mutation occurs on the matrix with probability 1/15. The magnitude *μ*_2_ is drawn from [−0.1/(15*N*^2^), 0.1/(15*N*^2^)] for each element *b*_*ij*_ independently, where *N* corresponds to the number of phenotypic traits.

### Evaluation of fitness

Following the framework used in [64], we define the fitness of the developmental system as a benefit minus cost function.

The benefit of a given genetic structure, *b*, is evaluated based on how close the developed adult phenotype is to the target phenotype of a given selective environment. The target phenotype characterises a favourable direction for each phenotypic trait and is described by a binary vector, *S* = 〈*s*_1_, …, *s*_*N*_〉, where *s*_*i*_ ∈ {−1, 1}, ∀*i*. For a certain selective environment, *S*, the selective benefit of an adult phenotype, *P*_*a*_, is given by (modified from [25]):

$$b=w\left(P_{a},S\right)=\frac{1}{2}\left(1+\frac{P_{a}\cdot S}{N}\right),$$ (1)

where the term *P*_*a*_ ⋅ *S* indicates the inner product between the two respective vectors. The adult phenotype is normalised in [−1, 1] by *P*_*a*_ ← *P*_*a*_/(*τ*_1_/*τ*_2_), i.e., *b* ∈ [0, 1].

The cost term, *c*, is related to the values of the regulatory coefficients, *b*_*ij*_ ∈ *B* [65]. The cost represents how fitness is reduced as a result of the system’s effort to maintain and reproduce its elements, e.g., in *E. coli* it corresponds to the cost of regulatory protein production. The cost of connection has biological significance [27, 64–67], such as being related to the number of different transcription factors or the strength of the regulatory influence. We consider two cost functions proportional to i) the sum of the absolute magnitudes of the interactions, $c={∥B∥}_{1}=\sum_{i=1}^{N^{2}}|b_{i}j|/N^{2}$, and ii) the sum of the squares of the magnitudes of the interactions, $c={∥B∥}_{2}^{2}\;=\sum_{i=1}^{N^{2}}b_{ij}^{2}/N^{2}$, which put a direct selection pressure on the weights of connections, favouring sparse (*L*_1_-regularisation) and weak connectivity (*L*_2_-regularisation) respectively [68].

Then, the overall fitness of *P*_*a*_ for a certain selective environment *S* is given by:

$$f_{S}\left(P_{a}\right)=b-\lambda c,$$ (2)

where parameter *λ* indicates the relative importance between *b* and *c*. Note that the selective advantage of structure *B* is solely determined by its immediate fitness benefits on the current selective environment.

### Chi-squared error

The *χ*^2^ measure is used to quantify the lack of fit of the evolved phenotypic distribution $\hat{P_{t}}\left(s_{i}\right)$ against the distribution of the previously experienced target phenotypes *P*_*t*_(*s*_*i*_) and/or the one of all potential target phenotypes of the same family *P*(*s*_*i*_). Consider two discrete distribution profiles, the observed frequencies *O*(*s*_*i*_) and the expected frequencies *E*(*s*_*i*_), *s*_*i*_ ∈ *S*, ∀*i* = 1, …, *k*. Then, the chi square error between distribution *O* and *E* is given by:

$$\chi^{2}\left(O,E\right)=\sum_{i}\frac{{\left(O\left(s_{i}\right)-E\left(s_{i}\right)\right)}^{2}}{E(s_{i})}$$ (3)

*S* corresponds to the training set and the test set when the training and the generalisation error are respectively estimated. Each *s*_*i*_ ∈ *S* indicates a phenotypic pattern and *P*(*s*_*i*_) denotes the probability of this phenotype pattern to arise.

The samples, over which the distribution profiles are estimated, are uniformly drawn at random (see Estimating the empirical distributions). This guarantees that the sample is not biased and the observations under consideration are independent. Although the phenotypic profiles here are continuous variables, they are classified into binned categories (discrete phenotypic patterns). These categories are mutually exclusive and the sum of all individual counts in the empirical distribution is equal to the total number of observations. This indicates that no observation is considered twice, and also that the categories include all observations in the sample. Lastly, the sample size is large enough to ensure large expected frequencies, given the small number of expected categories.

### Estimating the empirical distributions

For the estimation of the empirical (sample) probability distribution of the phenotypic variants over the genotypic space, we follow the Classify and Count (CC) approach [69]. Accordingly, 5000 embryonic phenotypes, *P*(0) = *G*, are uniformly generated at random in the hypercube [−1, 1]^*N*^. Next, each of these phenotypes is developed into an adult phenotype and the produced phenotypes are categorised by their closeness to target patterns to take counts. Note that the development of each embryonic pattern in the sample is unaffected by development of other embryonic patterns in the sample. Also, the empirical distributions are estimated over all possible combinations of phenotypic traits, and thus each developed phenotype in the sample falls into exactly one of those categories. Finally, low discrepancy quasi-random sequences (Sobol sequences; [70]) with Matousek’s linear random scramble [71] were used to reduce the stochastic effects of the sampling process, by generating more homogeneous fillings over the genotypic space.

## Supporting information

S1 Appendix Supplementary material. (PDF)
