# Supplementary material for: How evolution learns to generalise: Using the principles of learning theory to understand the evolution of developmental organisation
Source: PLoS Comput Biol. 2017 Apr 6;13(4):e1005358. doi: 10.1371/journal.pcbi.1005358 (PMC5383015; doi:10.1371/journal.pcbi.1005358)
Supplement: S1 Appendix — (PDF) [file pcbi.1005358.s001.pdf]

How evolution learns to generalise: Using the principles of learning theory to understand the evolution of developmental organisation.

Kostas Kouvaris[1,*], Jeff Clune[2], Loizos Kounios[1], Markus Brede[1], Richard A. Watson[1]
1 ECS, University of Southampton, Southampton, UK
2 University of Wyoming, Laramie, Wyoming, USA
∗ E-mail: kk6g11@soton.ac.uk

Supporting Information

Supporting Figures

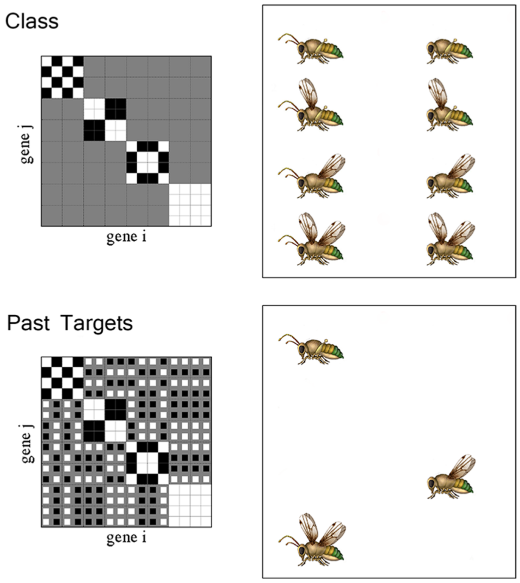

Fig A. The underlying correlational structure of the class and the training set. (Top) Hinton diagram of the variance-covariance matrix and phenotypic distribution of all potential future phenotypic targets. The true underlying structure of the given problem set which is comprised of all 8 possible phenotypic targets is described by the block diagonal interaction matrix. Accordingly, the traits within each module that encode for each functional part of the organism (e.g., front wings) are strongly correlated with each other (positively or negatively depending on the combination of signs in the particular phenotypic pattern used), and no correlations between one module and another (e.g., the production of halteres is functionally independent from the production of front wings). (Bottom) Hinton diagram of the variance-covariance matrix and phenotypic distribution of past phenotypic targets. The structure of the training set which is comprised of 3 phenotypic targets is described by an interaction matrix with non-zero off-diagonal elements. Those elements correspond to spurious correlations that describe functional phenotypic dependencies between modules that are present in the past selected phenotypic targets (e.g., the production of front wings is positively correlated with the production of antennas). Such developmental structures will appropriately represent the 3 past selected targets, but fail to generate all 8 phenotypes from the class. The colour and the size of the squares in Hinton's representation indicate the sign and the magnitude of the respective correlations.

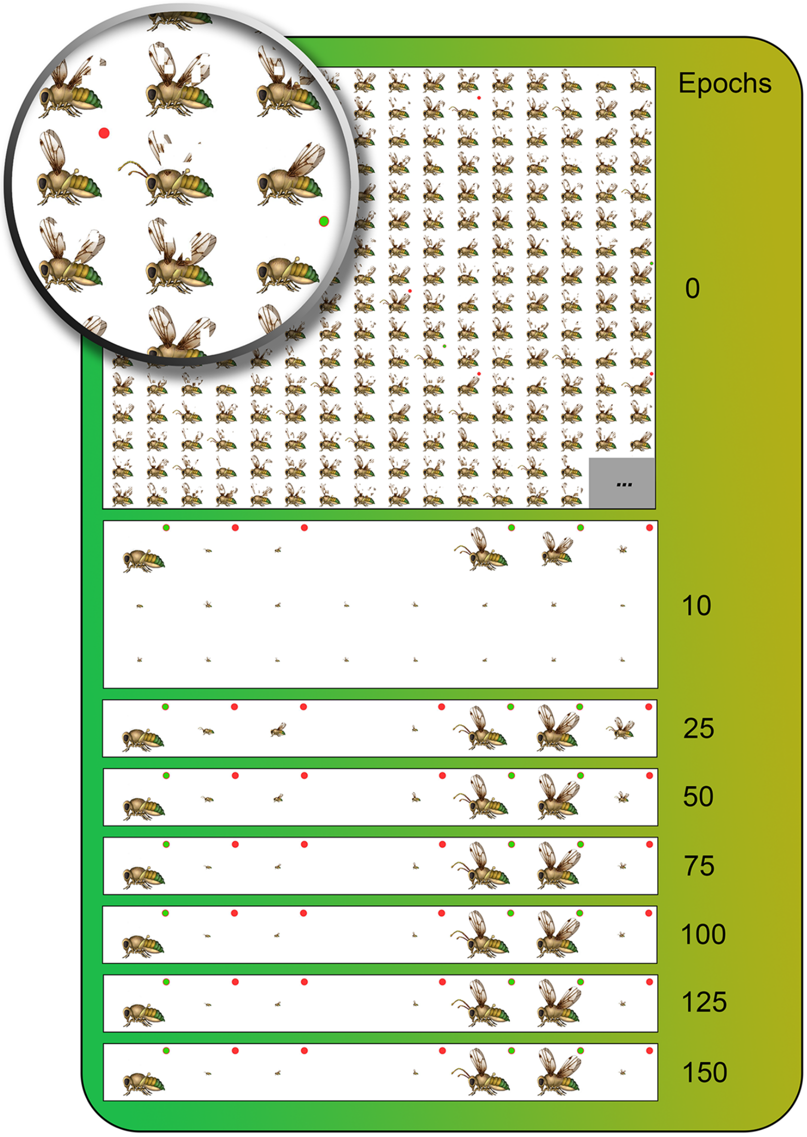

Fig B. The evolution of phenotypic distribution for moderate environmental switching. Pictorial representation of the phenotypic distributions induced by the evolving developmental process over evolutionary time for moderate environmental switching. Green circles indicate past selected targets, while red circles indicate previously-unseen phenotypes from the same phenotype family as the past ones. Phenotypes outside of the class are represented by distorted mosaic images. The size of the insect-like creatures indicates the propensity of development to express the respective phenotype. At the beginning (epoch 0), development equally predisposes the production of all possible phenotypic patterns (here 2^{12}), i.e., no developmental biases. The evolving developmental structure initially starts canalising only phenotypes from the class. After epoch 25 however it further canalises the production of past selected phenotypes, by reducing the propensity of producing those phenotypes from the class that were not selected in the past, i.e., over-fitting.

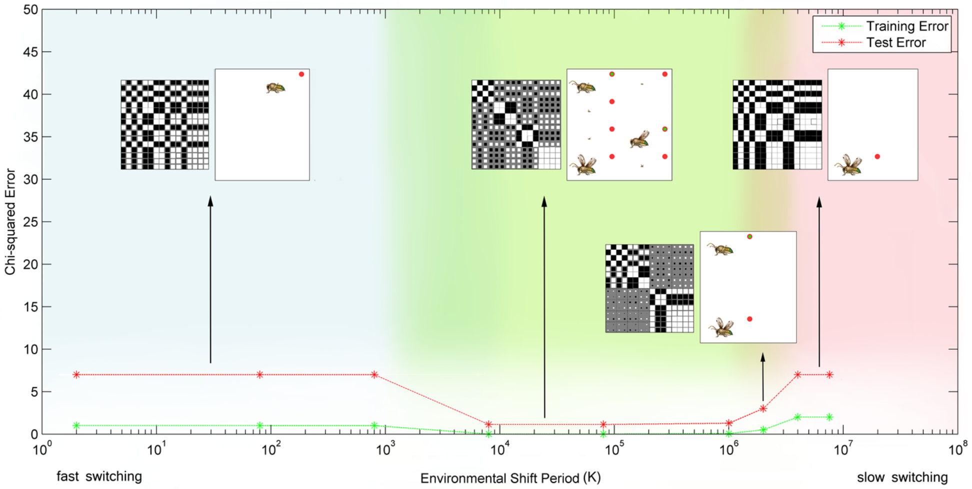

Fig C. Fast and Slow Environmental Switching Fail to Evolve Developmental Memory. The match between phenotypic distributions and the selective environments the network has been exposed to (training error) and all selective environments (generalisation error) against different environmental switching intervals (K). The insets illustrate the Hinton diagram of the evolved interaction matrix for each regime (indicated by different background colour) and the respective phenotypic distribution induced by the evolved developmental process.

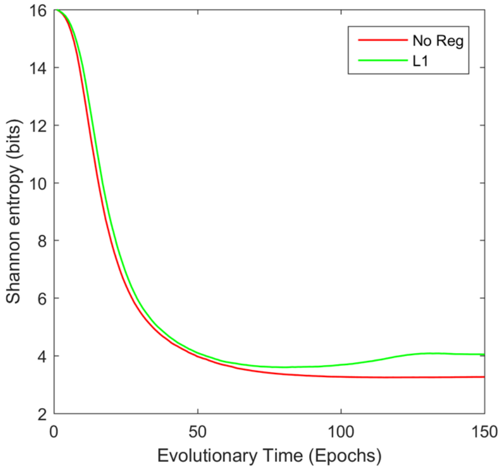

Fig D. Entropy of the phenotypic distribution reduces over evolutionary time. Shannon entropy [1] of the phenotypic distribution induced by the evolving developmental process for moderate environmental switching and sparse connectivity. Overfitting is indicated by reducing to less than four bits. For the case of sparse connectivity entropy converges to four bits indicating that each of the four modules vary independently. The sample size was 5×10^5.

Developmental Model

Following previous work [2], we describe the development of the embryonic phenotype to an adult phenotype by a continuous, non-linear and recurrent (i.e., it allows for feed-back connections) model of gene-regulatory networks [3, 4].

At each developmental time step, t, the phenotype of an individual organism is characterised by a collection of phenotypic traits, $P_t = \langle p_{t,1}, \ldots, p_{t,N} \rangle$, where $p_{t,i} \in \mathbb{R}, \forall i$. The genotype is comprised of two parts: the direct effects on the embryonic phenotypic traits, $G_t = \langle g_{t,1}, \ldots, g_{t,N} \rangle$, where $g_{t,i} \in \{-1, 1\}, \forall i$ and the regulatory interactions between the genes, b_{ij}, that determine the dynamical developmental process [5, 15, 16]. The regulatory interactions are represented by the matrix B.

The dynamics of the expression level for each gene depend on 1) the gene expression levels of the genes that is connected to and 2) the its pattern of connections, i.e., how strongly the respective gene is connected to its neighbouring genes. In the first time step, the embryonic phenotype is solely characterised by the direct effects of G ($P_0 = G$). Thereafter, at every developmental step the phenotypic traits are developed under the following set of difference equations [2, 8]:

$$p_{t+1,i} = p_{t,i} + \tau_1 \sigma(\sum_j b_{ij} p_{t,j}) - \tau_2 p_{t,i}, \tag{1}$$

where $\tau_1 = 1$ and $\tau_2 = 0.2$ indicate the maximal expression rate and the constant rate of degradation of the given gene product respectively. The second term in the right-hand side of equation (1) corresponds to the interaction term, the activity of which is limited by a non-linear, monotonic and bounded (sigmoid) activation function, $\sigma(x) = tanh(\alpha x)$, where $\alpha = 0.5$. Then, over a fixed number of developmental time steps, T (here $T = 10$), the embryonic phenotype is transformed into an adult phenotype, $P_a = P_T$, upon which selection can act. Both G and B are initialised at zero.

Varying Selective Environments

In this work, a set of related phenotypic targets is considered from the same family (as in [2, 9]). This guarantees that the environment changes in a systematic manner (i.e., shares common regularities invariant over time) — something which is ubiquitous in natural environments.

Since we are interested in modelling phenotypic variability, traits that are under constant selection are omitted from our model. We choose a simple family of modularly-varying targets. Modularity is widespread in the natural world and provides a simple way to test for generalised developmental oragnisations that are biological relevant [10–16]. For simplicity, to model selection that varies in a modular manner, we assume an extreme form of modularity, namely separable modules [17]. Accordingly, selection on any trait is strongly interdependent with selection on other traits in the same module, but independent of selection on traits in other modules. Specifically, when a change in the environment occurs, if the direction of selection on a given trait changes, the direction of selection on all other traits in the same module also changes (this defines the modules). Selection thus favours two complementary states for each module that confer high fitness in different environments. Since the selection on each module is independent of selection on other modules, this means that there are 2^k possible high-fitness phenotypes, where k corresponds to the number of modules.

Here we assume a class of phenotypes consisted of equal sized modules (4 modules of 4 phenotypic traits each). The particular patterns chosen are irrelevant. So we pick one phenotype of 16 traits arbitrarily, here $(-+-+--++-++-----)$, and divide it into 4 equal modules (i.e., $(-+-+)$, $(--++)$, $(-++-)$ and $(----)$). Accordingly, for the phenotypic patterns that belong in the class, each module (block) can have 2 states: A or B; denoting a particular phenotypic sub-pattern or sub-goal (e.g., here the sub-goal for the first module can be either $(-+-+)$ (A) or $(+-+-)$ (B)). The class is thus comprised of 16 different modular patterns; all possible combinations of the sub-patterns (blocks) (see Fig A in S3 Appendix).

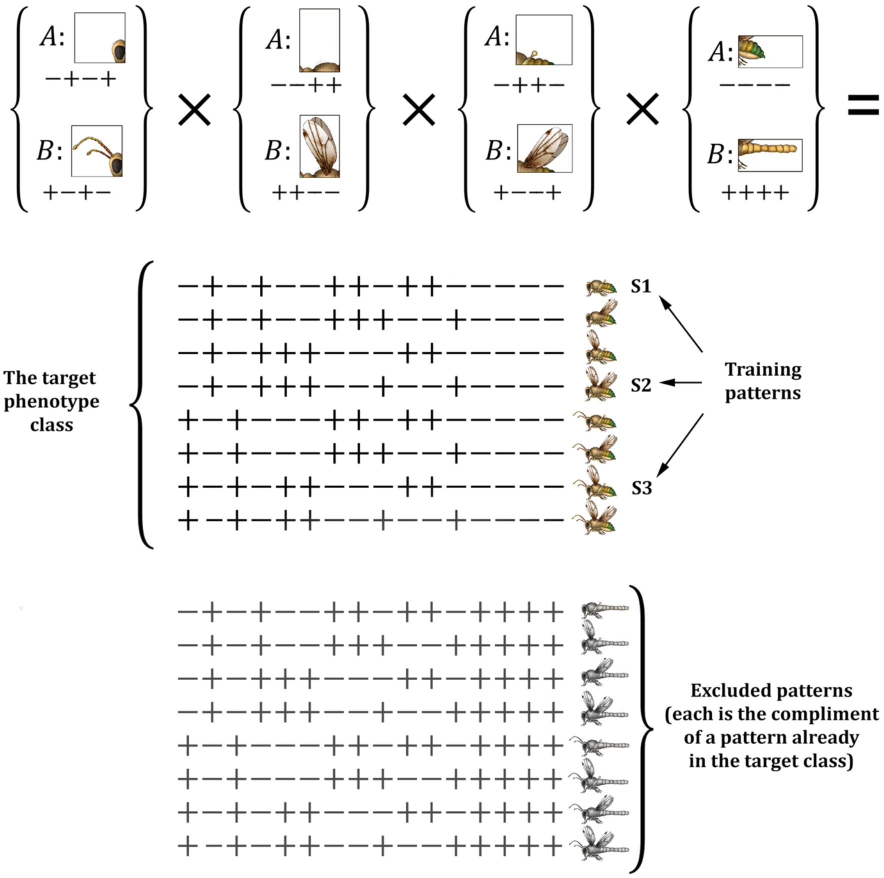

Fig A. Modularly-varying environment. Target phenotypes varying from one another in a modular fashion. Each target phenotype consists of 4 modules of 4 phenotypic traits (i.e., 16 phenotypic traits in total). Each module can take two (complementary) states: A or B; denoting particular sub-patterns favoured by selection in different selective environments. The complete set of phenotypes is thus comprised of $2^4 = 16$ phenotypes, differing from one another in a modular fashion. The signs of phenotypic traits correspond to the direction favoured by selection in a given environment. Eight of the 16 possible phenotypes are designated as the target class (the other eight are merely the complement of a pattern already in the target class). For the main experiments, three patterns from the target class are used as 'training' patterns, i.e., selected for.

The time-invariant regularities here are the correlations between traits within any one module. The actual underlying structure of the given problem can thus be described by the block diagonal interaction matrix (see Fig A in S1 Appendix). The colour and the size of the squares in Hinton's representation indicate the sign and the magnitude of each correlation respectively. This clearly shows that selection on the traits within each module are strongly correlated with each other (positively or negatively depending on the combination of signs in the particular phenotypic pattern used), and no correlations between one module and another.

Complementary patterns here are also stable states of the evolved dynamical system as a result of Equation 1. The map described in Equation 1 is an odd function (i.e., symmetric with respect to the origin) since $f(-x) = -f(x)$. Accordingly, if R is a stable state of the system, i.e., $R = f(R)$, then $-R$ is also stable since $-R = -f(R) = f(-R)$. In order to focus on the more interesting (non-trivial) attractors that may arise, we limit the phenotypic space so as to ignore complementary targets (i.e., thus removing 8 of the patterns). Specifically, without loss of generality, we consider the phenotypic targets in which the sub-pattern in the last slot (trait positions:$13-16$) corresponds to state A: $\{-,-,-,-\}$, i.e., we focus on the top-half of the class as arranged in the lower part of Fig 1. Accordingly, each member of the other half of the class is the bit-wise complement of a member in the top half.

In this work, we want to examine the ability of the developmental system to 'learn' from past selective environments and generalise to new environments by producing novel phenotypes within the same class. Accordingly, to assay generalisation and the conditions that promote it, the population is evolved by exposure to a limited number of selective environments (< 8, i.e., a strict sub-set of the class). Otherwise, generalisation would not be relevant, since the population would have been exposed to all possible selective environments (i.e., all phenotypes in the class are presented). For this paper, we use the following example from this problem domain as a training set:

$$\begin{aligned} S_1 &= \{-,+,-,+\},\{-,-,+,+\},\{-,+,+,-\},\{-,-,-,-\}. \\ S_2 &= \{-,+,-,+\},\{+,+,-,-\},\{+,-,-,+\},\{-,-,-,-\}. \\ S_3 &= \{+,-,+,-\},\{+,+,-,-\},\{-,+,+,-\},\{-,-,-,-\}. \end{aligned} \tag{2}$$

In Favouring Sparse Connectivity in Different Training Sets, we explore sensitivity to this particular choice by examining generalisation from training on all possible proper subsets of the class.

The Structure of Developmental Organisation

Here we show how costly interactions and noisy environments facilitate the emergence of more general and parsimonious developmental models. For this purpose, we monitor the evolution of regulatory interactions over evolutionary time in each evolutionary setting. The regulatory coefficients here correspond to the free parameters of the developmental model that determine the functional organisation of development.

We first analyse the evolution of regulatory coefficients in the control scenario, i.e., moderate rate of environmental change. Fig A A in S4 Appendix shows that the ontogenetic interactions evolved under natural selection to reflect the correlations in the previously-experienced selective environments. As seen, the Hinton diagram of the evolved regulatory matrix appropriately matched the variance-covariance matrix of the past phenotypic targets (Fig A in S4 Appendix). The colour and the size of the squares in Hinton's representation indicate the sign and the magnitude of the respective correlations.

Yet natural selection did not directly select either *for* correlations, or *for* matching the exploration distribution to the fitness distribution of the phenotypic variants (i.e., training error minimisation). Natural selection selected *for* immediate fitness differences depending on how well adapted the organism was to its current selective environment; i.e., how close the produced adult phenotype was to the respective target phenotype. The evaluation of the developmental process performed here against the training and the test set was a post hoc analysis, and hence not part of the actual evolutionary dynamics.

In the same fashion as the nervous system [18], evolution does not try to analyse anything. It just tries to generate appropriate behaviour. The observed (correlation) learning behaviour of evolution can be seen as a by-product of developmental systems' effort to produce high-fitness phenotypic variants in varied selective environments — optimise the actual functionality of the system. The system does not explicitly aim at inferring the target function, namely, the ideal G-P map that gives rise to proper system functionality in long-term (over certain genetic and environmental conditions). Nevertheless, we see that under certain conditions the system may discover a hypothesis (i.e., set of regulatory coefficients) closer to the target function, by producing phenotypic variants that are fitter in short term.

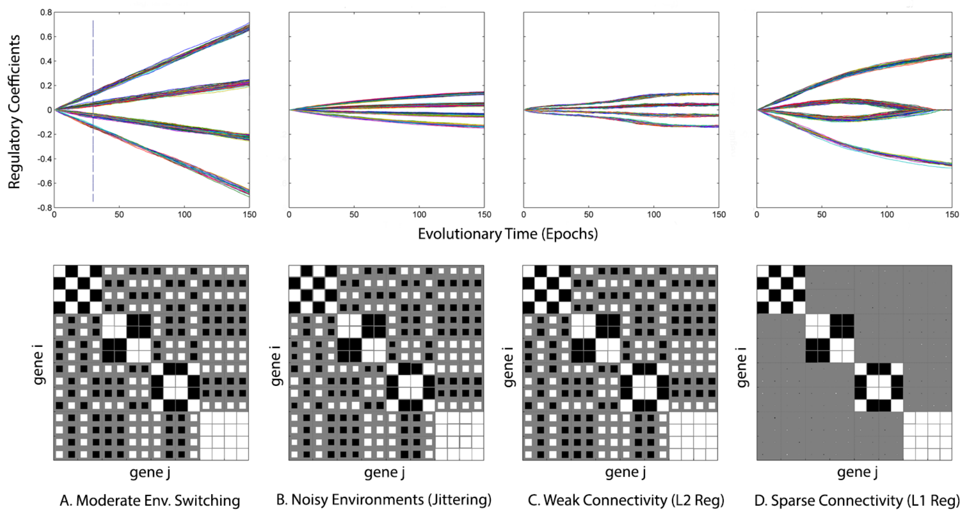

Fig A. Evolution of regulatory coefficients in noisy environments and under parsimony pressure. The evolution of regulatory coefficients over evolutionary time and the Hinton diagram of the evolved regulatory coefficients (after epoch 150) for (A) moderate environmental switching, (B) noisy environments, (C) favouring weak connectivity and (D) favouring sparse connectivity. The vertical dashed line denotes when the ad-hoc technique of early stopping is used, i.e., the moment the problem of over-fitting begins. Favouring sparsity ignores the weak spurious correlations of the finite sampling noise and maintains the time-invariant ones.

Fig A B in S4 Appendix shows that under the presence of environmental noise, the regulatory interactions evolved towards smaller in magnitude weights. In particular, we observe that the rate of evolutionary change was decreased with evolutionary time giving rise to a plateau in the test error in Fig 3 B. The set of evolved regulatory coefficients here corresponds to the one we get if we stopped evolution the moment over-fitting begins, i.e., at the vertical dashed line in Fig 3 A. From Hinton diagram we can see that the relative importance between strong and weak correlations remained the same as in the case of the control run, i.e., only the magnitudes changed. Therefore, noise had a beneficial role on the evolution of genetic structures by making it difficult for natural selection to find configurations that over-fit past phenotypic targets.

We observe similar results for the evolution of regulatory interactions under the pressure for weak connectivity (Fig A C in S4 Appendix). In contrast to environmental stochasticity, however, favouring weak connectivity imposes strict constraints on the evolution of regulatory coefficients that prohibit them from growing bigger, i.e., providing a hard bound determined by the strength of parsimony pressure (see below). Accordingly, the regulatory coefficients initially increased until they reached a level that the further increase in the reproduction and maintenance cost of interactions was greater than the benefit of the developmental structure. Moreover, when properly tuned favouring weak connectivity exhibits the same behaviour as stopping early. Favouring weak connectivity (L_2-regularisation) can be understood as imposing inductive biases (i.e., additional constraints) in the evolution of regulatory interactions, punishing interactions (parameters) with extreme (high) magnitudes by applying a penalty proportional to their current magnitudes (as in weight-decay).

Lastly, Fig A D in S4 Appendix illustrates how favouring sparse connectivity can exhibit a form of feature selection emphasising the relative importance of the strong correlations against the weak correlations. Specifically, we see that only the strongest (time-invariant) correlations persisted, while

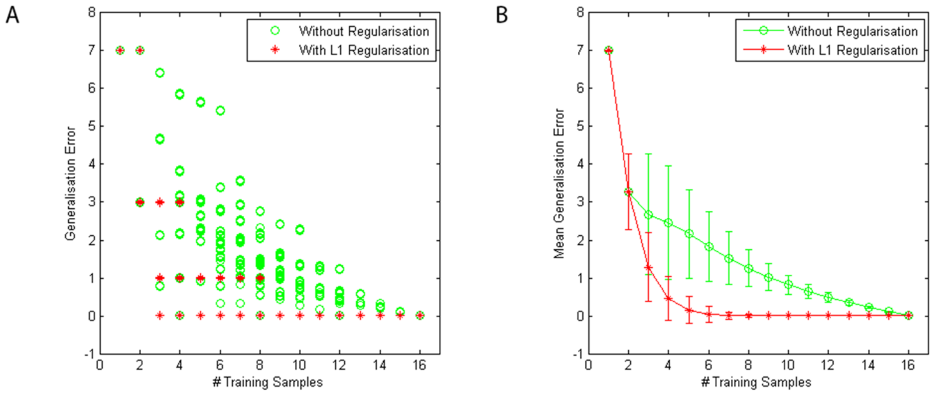

Fig A. Favouring sparse connectivity enhances phenotypic generalisation. (A) Phenotypic generalisation with and without the parsimony pressure for sparsity (L_1-regularisation) against all possible evolutionary scenarios (training sets), i.e., all possible combinations of distinct past selective environments drawn from the class. (B) Means and error bars of the generalisation performance of the evolved networks with and without the parsimony pressure for sparsity against different numbers of previously experienced selective environments. The cost of connection significantly enhanced evolvability in the majority of the cases. The interaction matrices here were determined using Hebb's rule.

the weak (spurious) correlations, which arose as a result of the sampling process, were eliminated over evolutionary time. The strong correlations here (i.e., the block diagonal of the interaction matrix) correspond to the actual underlying modular structure of the environmental variation that remain invariant over time. Consequently, if the strength of parsimony pressure is large enough to ignore the spurious correlations, the evolved associations are (almost) identical to the variance-covariance matrix that describes the phenotypes family (see Fig A in S4 Appendix). Favouring sparse connectivity (L_1-regularisation) can be understood as punishing interactions by equally applying a fixed penalty to all of the weights of the network. The amount of reduction is controlled by the hyper-parameter λ (see below); the higher its value, the higher the penalty applied, and hence the higher the level of sparsity. When properly tuned, favouring sparse connectivity leads to many zero weights, and thus the complexity of the model is reduced by removing degrees of freedom.

Favouring Sparse Connectivity in Different Training Sets

Experiments were also carried out for every possible training set as a strict sub-set of the test set. Firstly, all possible combinations, $\sum_{0\leq k\leq N}\binom{N}{k}=2^N$, were explicitly enumerated, where N indicates the number of patterns in the test set. Then, the respective developmental systems were determined following Hebb's rule with and without the selective pressure on the cost of connections (for optimal λ values). Hebbian learning was used here for computational tractability (65536 possible combinations), since it has been shown before that the interaction matrix evolves under natural selection in a Hebbian manner [2]. According to Hebb's rule, the pair-wise interactions are increased (or decreased) if the phenotypic traits are aligned (or not). The Hebbian matrix can be computed by computing the outer-product over the training inputs, i.e., the auto-correlation matrix. For the sake of comparison, the respective coefficient matrices were also tuned to be of the same average magnitude level as in the experiments above. These simulations allow us to draw some more general conclusions.

Overall, we find that the cost of connection significantly enhanced evolvability in the majority of the cases (Fig A in S5 Appendix). As the number of observations is increased we observe an increase

on average in evolvability, reaching zero generalisation error when $k = N$, even without incorporating the cost of connection. Interestingly, this was also true for some cases of 4, 8 and 12 patterns. We therefore see that different training sets entailed different information about the class, some of which were better representatives than others. For training sets consisted of more than half of the patterns in the class, we also observe that (optimally tuned) parsimony pressure for sparsity certainly resulted in perfect generalisation. On the other hand, in situations like the ones of 1 or 2 patterns the parsimony pressure had no effect on the generalisation performance of the network, and in some situations between 3 to 8 patterns it had little effect.

Acknowledgments

No data sets are associated with this publication.

References

1. Shannon CE. A mathematical theory of communication. ACM SIGMOBILE Mobile Computing and Communications Review. 2001;5(1):3–55.

2. Watson RA, Wagner GP, Pavlicev M, Weinreich DM, Mills R. The Evolution of Phenotypic Correlations and Developmental Memory. Evolution. 2014;68(4):1124–1138.

3. Vohradský J. Neural model of the genetic network. Journal of Biological Chemistry. 2001;276(39):36168–36173.

4. Vohradský J. Neural network model of gene expression. The FASEB Journal. 2001;15(3):846–854.

5. Wagner GP. The biological homology concept. Annual Review of Ecology and Systematics. 1989; p. 51–69.

6. Lipson H, Pollack JB, Suh NP. On the origin of modular variation. Evolution. 2002;56(8):1549–1556.

7. Kashtan N, Mayo AE, Kalisky T, Alon U. An analytically solvable model for rapid evolution of modular structure. PLoS computational biology. 2009;5(4):e1000355.

8. Wessels LF, van Someren EP, Reinders MJ, et al. A comparison of genetic network models. In: pacific Symposium on Biocomputing. vol. 6; 2001. p. 508–519.

9. Parter M, Kashtan N, Alon U. Facilitated variation: how evolution learns from past environments to generalize to new environments. PLoS Computational Biology. 2008;4(11):e1000206.

10. Clune J, Mouret JB, Lipson H. The evolutionary origins of modularity. Proceedings of the Royal Society b: Biological sciences. 2013;280(1755):20122863.

11. Callebaut W, Rasskin-Gutman D. Modularity: understanding the development and evolution of natural complex systems. MIT press; 2005.

12. Carroll SB. Chance and necessity: the evolution of morphological complexity and diversity. Nature. 2001;409(6823):1102–1109.

13. Alon U. An introduction to systems biology: design principles of biological circuits. CRC press; 2006.

14. Wagner GP, Pavlicev M, Cheverud JM. The road to modularity. Nature Reviews Genetics. 2007;8(12):921–931.

15. Lipson H, Pollack JB, Suh NP. On the origin of modular variation. Evolution. 2002;56(8):1549–1556.

16. Kashtan N, Mayo AE, Kalisky T, Alon U. An analytically solvable model for rapid evolution of modular structure. PLoS computational biology. 2009;5(4):e1000355.

17. Watson RA. Compositional evolution: the impact of sex, symbiosis and modularity on the gradualist framework of evolution. Mit Press; 2006.

18. Anderson JA. Cognitive and psychological computation with neural models. Systems, Man and Cybernetics, IEEE Transactions on. 1983;(5):799–815.
